# Intestinal Microbiotas and Alcoholic Hepatitis: Pathogenesis and Therapeutic Value

**DOI:** 10.3390/ijms241914809

**Published:** 2023-09-30

**Authors:** Jiazhen Zheng, Ziyi Li, Hengyi Xu

**Affiliations:** 1 Queen Mary School, Jiangxi Medical College, Nanchang University, Nanchang 330006, China; vozher@163.com (J.Z.); lzy15006333008@163.com (Z.L.); 2 State Key Laboratory of Food Science and Resources, Nanchang University, Nanchang 330047, China

**Keywords:** alcoholic hepatitis, intestinal microbiotas, pathogenesis, target, therapeutic potential

## Abstract

Alcoholic hepatitis (AH) is a rapidly progressing and severe stage of alcoholic liver disease, presenting a grim prognosis. Extensive research has elucidated several underlying mechanisms that contribute to the development of AH, including metabolic alterations, immune stimulation, and intestinal dysbiosis. These pathological changes intricately intertwine during the progression of AH. Notably, recent studies have increasingly highlighted the pivotal role of alterations in the intestinal microbiota in the pathogenesis of AH. Consequently, future investigations should place significant emphasis on exploring the dynamics of intestinal microbiota. In this comprehensive review, we consolidate the primary causes of AH while underscoring the influence of gut microbes. Furthermore, by examining AH treatment strategies, we delineate the potential therapeutic value of interventions targeting the gut microbiota. Given the existing limitations in AH treatment options, we anticipate that this review will contribute to forthcoming research endeavors aimed at advancing AH treatment modalities.

## 1. Introduction

Alcohol is a prevalent causal factor for chronic diseases globally, and its impact on liver disease development cannot be disregarded [1,2]. The effects of alcohol on the liver are intricate and multifaceted. Liver injury resulting from alcohol consumption can be classified into alcoholic liver disease (ALD), alcoholic fatty liver, alcohol-induced liver fibrosis, as well as acute and chronic inflammatory responses in the liver. These conditions progressively worsen from initial simple steatosis as alcohol intake and associated injuries accumulate. Ultimately, they can lead to severe cirrhosis, hepatic failure, or even hepatocellular carcinoma, making alcohol one of the leading causes of death among patients with liver disease [3,4].

**Figure 1:**
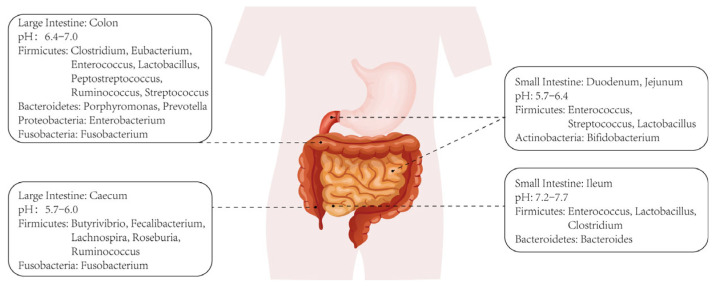
Classification and distribution of intestinal microbiotas.

A series of pathological changes occur in the liver, including progressive inflammation, hepatocyte necrosis, stellate cell activation-induced fibrosis, and disorders of lipid metabolism and cholestasis, which collectively form the underlying pathology of ALD [5]. Alcoholic hepatitis (AH), a specific pathology within ALD, typically exhibits a poor prognosis and high mortality rate. The etiology of ALD has been extensively investigated, revealing several contributing factors such as hepatocyte apoptosis, persistent stellate cell activation leading to hepatic fibrosis, extensive hepatocyte steatosis, and bile metabolism disorders. As research on intestinal microbiota intensifies, increasing evidence sheds light on their specific role in the progression of ALD. Figure 1 depicts the distribution of intestinal microbiota across various sections of the intestinal environment, illustrating their diversity and composition. In fact, intestinal microbiota can both metabolize and be affected by alcohol. On one hand, certain bacterial populations in the intestines can metabolize alcohol into products like acetaldehyde and acetic acid, which continue to exert effects on the liver or other microbiota upon entering the circulation [6,7]. On the other hand, excessive alcohol intake also disrupts intestinal microbiota, leading to imbalances in gut ecology, including the depletion of specific microbial species and alterations in microbiota composition ratios [8,9].

Disruption of the intestinal barrier function and dysbiosis of the gut microbiota can result in increased permeability of the intestinal wall. Consequently, products from the gut flora, such as lipopolysaccharides (LPS) and short-chain fatty acids (SCFAs), along with certain bacteria, are able to enter the circulation through the compromised intestinal wall. This migration of microbial substances, metabolites, and cytokines via the portal system enables them to reach the liver, triggering metabolic disorders and immune responses within various liver cells, including Kupffer cells, stellate cells, and neutrophils. These cellular interactions ultimately lead to the development of liver fibrosis, steatosis, and cell death [10,11,12,13]. In recent studies, there has been a growing focus on the therapeutic potential of gut microbiota. Currently, the treatment options for AH remain limited, with corticosteroids being among the few effective therapeutic agents [14,15]. However, investigations into the role of gut bacteria and their products have shown promising prospects. Notably, metabolites produced by gut bacteria, such as SCFAs, have demonstrated involvement in the repair and regulation of various AH injuries. These include restoration of the intestinal barrier, promotion of hepatocyte regeneration, inhibition of oxidative stress and the hyperimmune response, as well as metabolic regulation [16,17,18,19,20]. The findings from these studies provide valuable insights and open up new avenues for further exploration in the treatment of AH. Moreover, they inspire us to contemplate how we can leverage the therapeutic value of gut microbes to develop more precise, effective, and actionable interventions for AH patients, particularly when conventional treatment approaches are yielding diminishing effects.

In this review, we provide a comprehensive overview of the etiology, pathogenesis, and conventional treatment methods for AH. Additionally, we emphasize the significance of gut microbiota and their therapeutic potential in the development of AH. Our aim is to offer a fresh perspective that can guide future research endeavors in the field of AH treatment.

## 2. Pathological Process of Alcoholic Hepatitis and Related Microorganisms

### 2.1. Basic Pathological Process of Alcoholic Hepatitis

In the early stages of ALD, hepatic fat accumulation occurs, leading to the development of alcoholic fatty liver. This condition can then progress to AH and eventually advance to liver cirrhosis. Importantly, distinct molecular mechanisms are implicated at different stages of ALD, and their alterations often contribute to the malignant progression of the disease.

AH is characterized not only by neutrophil infiltration but also by acute alcoholic microvesicular steatosis (MIC), wherein affected hepatocytes may exhibit the presence of Mallory–Denk bodies [21,22,23]. Studies have indicated that the median age of AH patients is around 54 years [24]. In addition to prolonged heavy alcohol consumption and aging, there is evidence suggesting a genetic link to the development of AH. Research conducted on AH patients of European ancestry has shown that individuals carrying the PNPLA3 rs738409 polymorphism are at a higher risk of developing AH [25].

In addition to different subtypes of AH based on symptoms, severity can also vary among these subtypes. Acute AH and severe AH are particularly concerning due to their poor treatment outcomes and prognosis. Research has shown that severe AH carries a high mortality rate of 28% within 15 days [26]. However, it is important to note that non-severe AH should not be underestimated, as it still presents risks, with mortality rates of 6% and 13% within 28 days and 1 year, respectively [27]. Furthermore, elevated lipid levels, such as acylcarnitines, often serve as indicators of increased mortality in AH patients [28]. In severe cases, AH can progress to acute chronic liver failure, and the likelihood of death is greatly influenced by the extent of extrahepatic organ failure [29]. Although the precise pathogenic mechanisms of AH have not been fully elucidated, certain molecules have been associated with its development. Meagher et al. have proposed that heightened blood alcohol levels lead to cellular oxidative stress, which may contribute to AH progression [30]. Protein S can stimulate NKT cells in the liver, promoting AH [31]. Obesity can increase mortality in AH patients, possibly due to high CXCL11 expression caused by alcohol [32].

What is more, AH is a medical condition characterized by the abrupt or aggravated onset of jaundice and carries the risk of liver failure [33]. Brandl et al. have demonstrated that AH patients exhibit significantly elevated levels of fibroblast growth factor 19 (FGF-19) and serum bile acids (BA), both of which positively correlate with disease severity [34]. The increased presence of FGF-19 in biliary cells may contribute to the progression of AH towards hepatic fibrosis. Deregulation of biliary regenerative function in AH has been linked to heightened intrahepatocellular YAP levels [35]. In addition to their role in inflammation, neutrophils have been implicated in the damage of bile duct cells, leading to cholestasis, thereby challenging the previously held belief that cholestasis in AH solely results from liver cell injury [22]. Furthermore, AH is associated with the development of muscle atrophy.

### 2.2. Mechanisms of Microbial Intervention

#### 2.2.1. Dysbiosis of Intestinal Ecology

Gut microbes have been found to play a significant role in AH, as shown by studies utilizing shotgun metagenomics and polymerase chain reaction [7]. The abundance of bacteria in the gut changes during AH progression, with some bacteria increasing and others decreasing [36]. Changes in fungal metabolic pathways have also been observed in AH and may be associated with bacterial translocation in the gut [37]. However, it is important to note that a study found a limited correlation between changes in gut microbiota and the severity of AH [38]. Moreover, the pathogenesis of AH remains complex and requires further investigation. Several studies have suggested that dysregulation of the intestinal ecosystem due to chronic alcohol consumption and inflammation may be a significant contributing factor [39,40,41]. This dysregulation involves various physiopathological changes, such as compromised intestinal barrier function and increased permeability of the intestinal wall. These changes can occur due to alterations in the composition of the gut microbiota or migration of specific bacteria within the intestine.

Alcohol can induce liver damage indirectly by disrupting the intestinal barrier or by increasing intestinal wall permeability. Inflammatory factors, cellular debris, and even bacteria can then translocate through the intestinal barrier into the bloodstream and eventually reach the liver, triggering the injury process. Numerous pieces of evidence support the notion that gut microbiota play a crucial role in the progressive deterioration of gut barrier function [42,43]. Duan et al. have demonstrated that a high level of cytolytic *Enterococcus faecalis* is one of the significant characteristics of AH; moreover, the higher the concentration of bacteria, the higher the mortality rate of AH patients will be [44]. This provides support for the impact of gut microbiota changes on intestinal wall permeability. In addition, Lu et al. have found that viruses such as Herpesviridae, Parvoviridae, and Herpesviridae are increased in patients with AH [45]. Their findings further reinforce the conclusion that alterations in the composition of gut microbiota can contribute to the development of AH. Llopis et al. conducted a case study to examine the variations in intestinal microbial profiles among healthy individuals, patients with mild AH, and patients with severe AH. They proposed that the development of AH is closely linked to specific dysbiosis of the intestinal flora. The study revealed that Bifidobacteria and Streptococci were more abundant in patients with severe AH compared to healthy subjects, while Atopobium exhibited the opposite pattern. However, there was no significant difference in the abundance of these bacteria between healthy individuals and patients with mild AH. These findings suggest a specific association between Enterobacteriaceae and Streptococcaceae with the onset and severity of AH [46]. Numerous experimental results supported this direction, providing sufficient evidence for further exploration of how intestinal microbes are involved in the pathological process of intestinal ecological disorder in AH.

Many experiments suggest that metabolites produced by intestinal microbes may contribute to the damage observed in AH. In studies involving patients with early and advanced stages of ALD, researchers have observed increased permeability of the damaged intestinal wall to macromolecules, allowing toxic substances like endotoxins (LPS) to cross the intestinal barrier [47]. Prolonged exposure to LPS can lead to the disruption of Hypoxia Inducible Factor (HIF) in the intestinal epithelium of mice. HIF is known to play a crucial role in maintaining intestinal barrier function, promoting anti-inflammatory effects, and regulating important signaling pathways such as the synthesis of antimicrobial peptides in the mucosa. However, LPS can impair epithelial HIF-1α and HIF-2α, resulting in increased epithelial permeability [2,48]. This heightened state of permeability allows LPS to enter the circulation through the compromised barrier, triggering various reactions. It also facilitates the translocation of intestinal flora and their products to the liver, perpetuating a vicious cycle. Once in the circulation, LPS reaches the liver via the portal system and binds to astrocytes, Kupffer cells, and Toll-like receptor 4 (TLR4) on hepatocytes. This binding activates downstream signaling pathways, including NF-κB, leading to the release of inflammatory factors and initiating inflammatory responses and fibrosis in the liver [49,50].

Fascinatingly, altered gut ecology does not necessarily appear to be directly linked to severe AH pathology. Experiments conducted by Martino et al. have demonstrated that long-term chronic alcohol consumption in mice with humanized gut flora results in elevated blood acetate levels. Surprisingly, this leads to the initiation of intracellular acetate dissimilation rather than direct ethanol metabolism by intestinal microbes, which was previously believed to contribute to liver damage. Consequently, changes in blood acetate levels induce alterations in gut microbial composition. However, increasing acetate levels alone did not lead to further worsening of liver disease in mice, which may represent a more complex correlation between alcohol, the gut microbiome, and gut ecological deterioration that needs to be further explored [51].

#### 2.2.2. Metabolism

It is crucial to highlight the inherent overlap in the categorization of pathogenic mechanisms associated with intestinal microbiota in AH. This overlap stems from the logical correlation and coherence within the overall pathogenesis of the disease. The metabolism-related pathogenic mechanisms discussed in this section are intricately intertwined with the alterations observed in gut ecology, as described in the preceding section. By presenting these diverse logics in meticulous detail, we can gain a comprehensive understanding of the intricate interplay between gut microbiota, metabolites, intestinal barrier function, and liver damage in AH.

In the presence of alcohol intervention, there are significant alterations in hepatocyte metabolism. In AH, glucose metabolism in hepatocytes is affected, as evidenced by increased expression of hexokinase domain containing 1, leading to the accumulation of glucose-6-P and glycogen [52]. Furthermore, Glavind et al. have observed a decrease in urea synthesis in AH, which can serve as an indicator for evaluating disease severity [53]. Abnormalities in lipid metabolism, such as the occurrence of Acylcarnitines in AH, may be associated with high mortality rates [28]. Apart from changes in liver cell metabolism, there are also alterations in the metabolism of gut microorganisms. Interestingly, these changes in microbial metabolism do not necessarily align with corresponding changes in serum components. For instance, despite increased tryptophan synthesis by gut microbes in AH, serum tryptophan levels remain decreased [54]. This suggests that the influence of microbial metabolism on AH symptoms may be complex. Additionally, the biotin metabolism pathway of the gut microbiome is inhibited. Given that biotin serves as a cofactor for several carboxylases in mitochondria, this inhibition may contribute to mitochondrial damage in hepatocytes during AH [55].

Furthermore, alcohol-induced intestinal dysbiosis contributes to the development of metabolic disorders, which play a significant role in the pathological changes observed in AH. AH often coincides with impairment or alteration of the enterohepatic circulation pathway of BAs, which may be attributed to disturbances in intestinal ecology or alterations in gut microbiota. Studies conducted by Kakiyama et al. and Bajaj et al. have reported higher levels of secondary BAs in fecal samples from individuals with alcohol-related liver lesions, indicating increased hepatic–hepatic circulation of BAs. Significant dysregulation of intestinal ecology is also observed in some cases [56,57]. The research by Hartmann et al. provides robust evidence supporting the involvement of gut microbiota in the regulation of the BA cycle, and highlights the close association between changes in the intestinal environment, alterations in the microbial composition in ALD, and disruption of the BA cycle. In mice, decreased activity of farnesoid X receptor (FXR) receptors in the intestinal epithelium and increased expression of hepatic cholesterol 7α-hydroxylase (Cyp7a1) protein suggest that the changes observed in the disease progression are likely linked to the weakening of intestinal barrier function caused by metabolized secondary BAs [58]. Disorders in BA metabolism are likely to exacerbate the process of steatosis during AH and further contribute to the deterioration of the condition.

#### 2.2.3. Immunity

Several studies have shown a significant increase in macrophages in AH [59]. However, alcohol has been found to inhibit the phagocytic ability of macrophages and may contribute to the accumulation of bacteria in the liver [60]. Inflammatory factors play a crucial role in the development of AH. Cytokines such as TNF-α have been confirmed to increase in AH [41]. IL-20 can inhibit the production of the protective cytokine IL-6, exacerbating AH [61]. This suggests that the role of cytokines in alcoholic inflammation may not be direct. LPS, a product of intestinal flora, can enter the liver through the portal vein in the presence of alcohol-induced impairment of the intestinal barrier. LPS binds to TLRs on the surface of liver cells or immune cells, contributing to liver inflammation [62]. The LPS-TLR4 pathway is involved in AH patients and can stimulate the expansion of ductular cells and keratin expression, leading to portal hypertension [63]. Furthermore, not all immune responses worsen the progression of inflammation. The binding of TLR5 and bacterial flagellin is suggested to be associated with the protection of the intestine [64].

**Figure 2:**
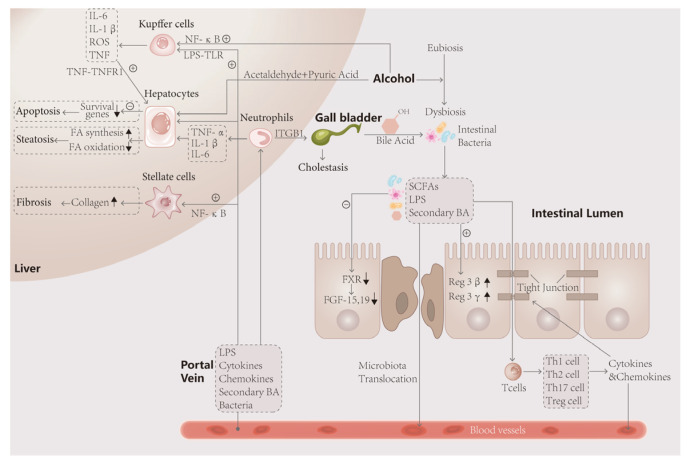
The intestinal flora is closely linked to the liver through the gut–liver axis. After ingestion of alcohol, the unmetabolized portion and acetaldehyde and acetic acid enter the intestine, disturbing the ecological stability of the intestinal lumen and causing disproportionality in the composition of the bacterial flora, such as an elevated Firmicutes/Bacteroidetes ratio, and metabolic disorders. The decrease in FXR gene expression and FGF-19 synthesis by intestinal wall cells, which have protective effects, promoted apoptosis of intestinal wall cells on the one hand, and the decrease in FGF-19 entering the liver, on the other hand, led to the impaired metabolism of bile acids, resulting in fatty infiltration of the liver and steatosis of hepatocytes. In turn, toxic products such as LPS leaking through the intestinal wall activate immune cells, leading to the secretion of large amounts of inflammatory factors that attack the tight junction and hepatocytes, leading to more severe weakening of the intestinal wall barrier and hepatocyte death. Activated neutrophils exacerbate the inflammatory response and attack bile duct cells, causing cholestasis and exacerbating the lesions.

In conclusion, microbial intervention has an important impact on the pathogenesis of AH. In the human environment, intestinal flora undergoes a series of metabolic and colony composition changes under the action of alcohol, which affect the intestinal tract, liver and other tissues through its metabolites, as shown in Figure 2.

## 3. Current Treatment of Alcoholic Hepatitis

Cortisol, a glucocorticoid (GC), has demonstrated a significant therapeutic effect in reducing mortality in AH and has been used for approximately 40 years [14]. While current therapies are not entirely effective, studies have shown that abstinence from alcohol can significantly reduce mortality compared to continued drinking [65]. In cases of severe AH that do not respond to glucocorticoid treatment, liver transplantation is often chosen as a therapy to reduce mortality [66,67]. In recent years, there has been increasing interest in targeted therapies that focus on the intestinal flora and associated inflammatory factors. Various substances can be used as therapeutic adjuvants to enhance the effectiveness of treatment. Besides these conventional treatments and care measures, promising therapeutic substances are being investigated.

### 3.1. Prednisolone

Prednisolone, a typical GC, is widely used in the treatment of severe AH. It has a good anti-inflammatory effect but primarily improves short-term mortality and is not highly effective for long-term treatment [68]. The effectiveness of GC therapy can be evaluated using the Lille score [69]. Additionally, studies have shown that the response to GC treatment is influenced by the extent of liver damage and whether abstinence from alcohol is practiced after treatment. Combination therapy involving prednisolone with other drugs is currently receiving significant attention. Research has demonstrated that combining prednisolone with S-adenosyl-L-methionine can enhance treatment effects compared to using prednisolone alone [70]. Furthermore, the therapeutic combination of prednisolone and N-acetylcysteine can improve the 1-month survival of severe AH patients [71]. However, the therapeutic effect does not significantly improve when combined with pentoxifylline, another drug used for AH treatment [72]. Thus, the mechanism of combination therapy involving prednisolone is complex. Prednisolone treatment also poses several challenges. The glucocorticoid receptor (GR) is affected in severe AH [73]. As prednisolone can bind to the mineralocorticoid receptor and cause adverse effects, it is important to design more effective drugs that specifically target the GR. Moreover, the use of prednisolone for severe AH treatment carries an increased risk. Studies have shown that prednisolone use can elevate the risk of infection [74]. Testing the level of bacterial DNA in the blood before treatment can help guide subsequent treatment decisions [74].

### 3.2. Liver Transplantation

For patients who do not respond to steroid therapy, liver transplantation is considered as another treatment option. According to research, patients who undergo early liver transplantation have relatively high 1-year (94%) and 3-year (84%) survival rates [75]. However, liver transplantation does not guarantee a favorable prognosis. The presence of infections during the treatment process increases the difficulty of treatment to some extent. Another simulation estimate has shown that patients who continue to consume alcohol after liver transplantation have significantly reduced postoperative survival time [76]. Heavy alcohol consumption after transplantation can lead to re-damage of the transplanted liver. Therefore, maintaining abstinence from alcohol is crucial when dealing with AH. Methylprednisolone use can exacerbate the adverse effects of liver transplantation [77].

### 3.3. Target Therapy

Riboflavin has been shown to inhibit AH mediated by ethanol [78]. Metadoxine has been found to improve the survival rate of patients with severe AH at three months and six months [79]. Furthermore, the expression of genes like Fmo5 and PPARα have a protective effect on ALD through the NF-κB signaling pathway [80]. Similarly, NLRP6 can inhibit NF-κB and protect the liver in AH [81]. These protective genes could serve as targets for future research. Ginsenoside Rg1 has been reported to reduce plasma transaminase and total bilirubin levels in AH patients while also decreasing inflammatory factors such as TNF-α and IL-3, indicating its protective role in AH by inhibiting oxidative stress and hepatocyte apoptosis [82]. Targeted therapies for specific gut flora have also been extensively studied. Bacteriophages designed to target cytolytic E. faecalis have been successfully developed [44]. Candidalysin, secreted by the commensal gut fungus Candida albicans, can damage hepatocytes in ALD, and it is being investigated as a potential target for disease treatment [83].

### 3.4. Complementary Therapeutic Substances

Moreno et al. have suggested that combination therapy with nutritional supplementation and corticosteroids is beneficial for AH treatment [84]. Therapeutic trials targeting substances like vitamin E have shown improvements in plasma markers in AH patients, although their impact on the disease itself may not be significant [85]. However, vitamin E and similar substances can be used as adjuvants to conventional treatment to enhance therapeutic effects [86]. Rifaximin has also demonstrated usefulness as an adjunct to glucocorticoids in the treatment of severe AH [87,88]. Injectable insulin combined with glucagon has shown potential for the treatment of acute AH, but it has not received widespread attention in recent years due to the development of updated treatment methods [89,90]. However, studies indicate that patients with acute AH who receive insulin injections instead of glucagon have lower mortality rates [89]. Furthermore, different treatment protocols may be necessary based on the severity of AH. Extensive research is still needed to develop effective therapies for AH in the future.

## 4. Role of Intestinal Microbiotas in Therapeutic Aspects

### 4.1. Probiotics and Fecal Microbiota Transplantation

As the most severe form of ALD, the prognosis for AH remains unfavorable despite current treatments. Given the significant regulatory influence of intestinal microbiota on liver metabolites and their involvement in essential liver metabolic processes, it is reasonable to consider the therapeutic potential of gut microbiota. However, the available precedents and clinical trials for gut microbiota therapies in alcoholic hepatitis are limited. Currently, treatment protocols mainly rely on the transplantation of healthy fecal flora and the use of probiotics, which are commonly employed in clinical practice. Thus, there is a need to shift our focus to the emerging field of AH therapeutic research on gut flora and explore its future therapeutic role through more precise and targeted therapies.

Recent years have witnessed a plethora of therapeutic applications involving recombinant editing of intestinal flora. The administration of probiotics, prebiotics, antibiotics, and healthy fecal transplants to “reshuffle” the body’s flora has shown promising potential for clinical application. Studies have revealed that adjusting the flora structure or restoring the original composition of intestinal flora can lead to improvements in intestinal ecology restoration, repair of the intestinal barrier function, and mitigation of hepatic oxidative stress damage [91,92]. Gupta et al. conducted a double-blind experiment, treating AH patients with the probiotics *Lacticaseibacillus rhamnosus* and *Lactobacillus helveticus*, and evaluated the efficacy of the treatment by monitoring the recovery time of intestinal flora and changes in the expression levels of liver injury markers. The results indicated an increase in the proportion of Bacteroidetes and a significant decrease in Proteobacteria and Fusobacteria after 7 days of treatment, suggesting that probiotic supplementation aids in restoring damaged intestinal flora in AH. Moreover, the treatment led to reduced expression of liver enzymes such as AST and ALT and a significant decrease in the levels of damage-causing LPS, indicating a positive effect of *Lacticaseibacillus rhamnosus* and *Lactobacillus helveticus* on improving liver function in AH [93].

Table 1 illustrates the therapeutic effects that can be achieved in AH by probiotics and other treatments that alter the composition of the gut microbiota.

Further experiments have demonstrated that intake of probiotic bacteria can ameliorate alcohol-related liver disease injury. Even severe conditions like alcoholic cirrhosis have shown improvement with appropriate treatment, such as the use of Lactobacillus casei Shirota, which effectively inhibits the expression of TNF receptor and IL-10 while restoring neutrophil function [95]. A pilot study conducted in 2017 by Philips et al. showcased the significant efficacy of fecal microbiota transplantation (FMT) in improving liver disease severity indices when implanting gut flora from a healthy population into the intestinal environment of patients with severe AH. Follow-up assessments revealed that patients with severe AH experienced enhanced survival rates and improvements in severe complications. Notably, Philips et al. also observed a shift in the relative abundance of gut flora similar to probiotic treatment. One year later, the patient’s flora composition became more aligned with that of the donor, characterized by a dominant presence of Firmicutes and changes in Proteobacteria and Actinobacteria abundances, which may have been influenced by donor modifications to the microbial community [96]. In a subsequent randomized comparative clinical trial, Pande et al. demonstrated that FMT is both effective and safe for treating patients with severe AH, comparing it to prednisolone treatment. The FMT group exhibited a significant survival advantage at both the 28-day and 90-day marks (F:P = 88.33%:78.33% and F:P = 75%:56.6%). Furthermore, the establishment of a new intestinal flora community through FMT reduced the incidence of infections, thus aiding in the restoration of the patients’ intestinal ecologies. This restoration, in turn, helped alleviate inflammatory responses, mitigate toxicities such as LPS substances reaching the liver, and promote liver damage repair [97].

Given that both probiotics and FMT serve as macro-regulatory tools, a more efficient treatment approach necessitates a precise exploration of their mechanisms of action. Further studies are required to investigate the changes in flora abundance and the intricate relationship between the liver–gut axis. Microbiome and metabolome level analyses, along with more targeted research, hold the potential to uncover these connections more comprehensively.

### 4.2. Therapeutic Potential and Future Prospects

In addition to the clinical trials demonstrating the effectiveness of gut flora and their products, there is a significant body of in vitro and animal experiments that highlight the involvement of gut flora and their products in repairing damage and halting disease progression in AH and other ALD pathologies. While these experiments cover a range of pathological stages and changes, they mainly focus on alcohol-related early damage leading to AH or hepatic pathological damage resembling AH-specific lesions. It should be noted that these experimental results do not directly establish the therapeutic role of gut flora. However, both the pathological features of the models used and the mechanisms by which gut bacteria exert their effects bear similarities to the features observed in AH and existing human studies.

It is worth emphasizing that in isolated cell and animal experiments, gut flora and their metabolites have shown positive effects in various ways on AH and other alcohol-related disease models. Nonetheless, it is important to acknowledge the notable differences between these isolated experiments and clinical studies that rely on the human environment. Consequently, the experimental results do not possess immediate clinical applicability.

Overall, the aim of summarizing these relevant experiments is to provide valuable insights for future research and development. By doing so, we can unveil potential research directions, aiding in the identification of targets and the development of innovative approaches for future clinical investigations. This, in turn, holds the promise of achieving more precise clinical strategies and significantly reducing the drug burden on patients throughout their treatment journey.

#### 4.2.1. Intestinal Barrier Protection

Gut microbes exert their most prominent effects within the environment they colonize, known as the gut lumen. In the context of AH, both alcohol consumption and intestinal inflammation contribute to the destruction of the intestinal environment and compromise the integrity of the gut barrier. This disturbance subsequently disrupts the growth and proliferation of bacteria within the gut, leading to liver damage through indirect pathways such as the circulation of inflammatory factors into the bloodstream and their subsequent impact on the liver. Interestingly, certain intestinal microbiotas possess the remarkable ability to effectively restore the damaged intestinal environment. Histological analyses have yielded results indicating that gut bacteria play a crucial role in maintaining intestinal homeostasis and preserving the integrity of barrier function. A recent study further suggests that the absence of gut bacteria, particularly due to decreased levels of Firmicutes and Lachnospiraceae, along with reduced production of the short-chain fatty acid (SCFA) butyrate via the acetyl-CoA pathway, is a key factor in the development and characterization of alcohol-induced liver injury and alcoholic liver disease [98]. Consequently, we conclude that preserving the integrity of the intestinal barrier serves as a suitable complementary approach to enhance the efficacy of targeted therapies. Restoring healthy microbiota structure is of great significance in reducing intestinal damage and restoring normal microbial composition.

In a study conducted by Sangineto et al., it was observed that the administration of Bacteroides Thetaiotaomicron in a mouse model of AH-related diseases resulted in a reduction in circulating LPS levels and a gradual recovery of intestinal mucosal thickness. The researchers further demonstrated that Bacteroides treatment increased the content of Mucin2, a main component of intestinal mucosal mucus, by downregulating the expression of Mucin1 and upregulating the expression of Mucin2. This effect helped alleviate the depletion of mucus caused by the consumption of an ethanol solution. Additionally, the downregulation of Claudin 1 induced by Bacteroides not only restored the expression levels of tight junction proteins, crucial for maintaining intestinal barrier function, but also inhibited the activity of the Notch pathway. This inhibition relieved the suppression of Mucin2 expression. Based on the similarity of the results of experiments using propionate to restore the intestinal mucosa, it is reasonable to infer that this restorative effect may have originated from the propionate produced by Bacteroides [99,100]. Li, H. et al.’s treatment of AH-related diseased mice with Lactobacillus resulted in a significant increase in acetic acid, propionic acid, and butyric acid in the intestine. The levels of serum ALT, AST, and LPS were decreased, while the expression of the constituent proteins of the tight junction in the ileum, such as ZO-1, claudin-1, and occludin, was higher compared with that in the control group. It can be seen that Lactobacillus has a significant restorative effect on intestinal barrier damage in alcoholic liver disease, and can inhibit an increase of intestinal wall permeability and improve the leakage of toxic substances into the circulation [101].

We summarize the results of nonclinical trials of the use of gut microbiota and its products in the treatment of alcohol-induced liver injury and disease and present the results in Table 2.

#### 4.2.2. Metabolic Regulation

Alcoholic liver disease (ALD) patients, including those with AH, typically experience a range of metabolic alterations. Hepatic steatosis and altered glucose metabolism are the most frequently observed metabolic problems in this patient population [121]. Therefore, studying the role of gut microbiota in alcohol-induced metabolic alterations is helpful in finding potential therapeutic targets.

Firmicutes and Bacteroidetes, as the most abundant microbiota in the intestines, play a crucial role in regulating liver metabolism. They contribute to maintaining liver health by influencing the host’s blood glucose and lipid levels through the regulation of sugar and lipid metabolism in the liver. Key substances involved in this regulation are SCFAs secreted by intestinal microbiota, particularly Firmicutes and Bacteroidetes [122,123]. Several experiments have demonstrated that short-chain fatty acids (SCFAs) play a significant role in regulating lipid metabolism in adipocytes and hepatocytes, particularly in cases of diet-induced obesity, lipid metabolism disorders, and nonalcoholic disorders. For instance, butyrate, secreted by Firmicutes, enhances the expression and transcription of the FGF-21 gene in hepatocytes by inhibiting HDAC3l. It also decreases peroxisome proliferator-activated receptor (PPAR)-α levels, leading to increased FGF-21 expression. The upregulation of FGF-21 promotes intracellular fatty acid oxidation, ketone body production, lipid consumption, and reduced hepatic steatosis [103]. This notion is supported by Zhu et al.’s observation that sodium butyrate upregulates thermogenic regulators Ucp-1 and Pgc-1α in mice, promoting thermogenic fat consumption [106]. Moreover, SCFAs can stimulate intracellular lipid β-oxidation in hepatocytes by inhibiting PPAR-γ expression, increasing mitochondrial uncoupling protein 2 expression and the AMP/ATP ratio, thereby activating AMPK [104,124]. Alternatively, they can activate GPR41 to raise intracellular calcium ion levels and consequently activate the lipid metabolism gene transcription factors CaMKII and CREB. This leads to the upregulation of leptin synthesis in adipose cells, which suppresses appetites in patients [107]. However, the effects and pathways of SCFAs in the context of AH are not yet well understood. Recently, Ding et al. discovered that Lactobacillus plantarum ZY08 effectively restored the composition of intestinal flora and increased SCFA production during probiotic treatment of mice with ALD-related diseases. The treatment reversed the expression of the lipid metabolism-related genes Cd36, Fasn, Dgat1, and Dgat, while PPAR-α gene levels remained relatively unchanged. However, the expression levels of related proteins significantly increased. Additionally, liver tissue section staining revealed a reduction in lipid droplets in the treatment group [109]. Nonetheless, there is a scarcity of comparative treatment experiments conducted in models that fully mimic the relevant pathological environment, necessitating further studies for validation.

AH can disrupt liver sugar metabolism. Prolonged alcohol consumption and inflammatory responses can damage β cells and induce insulin resistance in liver cells, leading to reduced glycogen synthesis capacity and impaired regulation of blood glucose levels, further exacerbating liver damage [125]. Both butyrate and propionate have the ability to regulate glucose metabolism. They modulate the expression of glycogen synthase genes and lipid metabolism in the liver, thereby controlling carbohydrate synthesis and maintaining stable blood glucose and lipid levels [126,127]. Propionate stimulates the secretion of the intestinal hormone GLP-1 by activating the GPCR41 receptor in intestinal L cells. This activation triggers downstream signaling pathways involving cAMP, calcium ions, and MAPK, ultimately upregulating GLP-1 expression [122,128,129].

Interestingly, however, some findings suggest that perhaps gut flora products are not as effective in the human body as we have envisioned and as isolated cell experiments have shown. The β-oxidation of lipids by SCFAs such as butyrate was not evident when precision-cut liver slices (PCLSs) modeled with adipose denaturation were used to replace the in vitro cultured cell models used in most experiments [130]. This has also led us to revisit the pathways by which the differences between pathology models and the real human environment act on gut flora products and their products. Perhaps addressing the interference of the multicellular environment is also a future aspect that needs to be addressed in the direction of the clinical application of intestinal flora therapy.

#### 4.2.3. Inflammation and Oxidative Stress Protection

Intestinal microbiotas play significant roles in the regulation of immune responses, primarily by inhibiting inflammatory reactions and providing protection against oxidative stress. In patients with AH, liver injury is largely attributed to endotoxin LPS generated by dysbiotic intestinal flora. LPS triggers the activation of the NF-κB pathway and induces the production of inflammatory mediators like TNF-α and IL-6, leading to oxidative damage in liver cells [131,132]. Certain intestinal microbiota, such as Bacteroidetes, can mitigate the inflammatory response in the liver through the secretion of SCFAs. Bacteroidetes, an anaerobic gram-negative bacterium lacking cell wall structure and spores, like its counterpart Firmicutes, particularly Thetaiotaomicron, produces SCFAs, including propionate and acetate, which exert hepatoprotective effects by reducing inflammatory factors and mediating modulation [110,133,134,135,136]. Propionate and butyrate, as SCFAs, can inhibit the production of inflammatory factors, such as the NF-κB transcriptional regulatory factor, thereby diminishing the inflammatory response in the liver and promoting liver health.

Previous studies have highlighted that SCFAs can modulate intestinal permeability, allowing inflammatory cytokines released from the intestinal lumen to enter the liver and cause injury [137]. In another experiment, SCFAs were observed to inhibit the activity of the NF-κB pathway by reducing the dimerization of NF-κB and p50 in intestinal cells [110]. Based on these findings, it is plausible to speculate that SCFAs may also participate in the regulation of the inflammatory response in the liver through a similar pathway. This hypothesis was further supported by the experiments conducted by Qi Xu et al., wherein AH-related diseased mice were treated with propionate, yielding positive results. The enhanced intestinal epithelial permeability in the mice was improved, effectively reducing intestinal inflammation and preventing the leakage of LPS into circulation. This, in turn, led to the inhibition of TLR4 receptor expression and downstream factors in liver Kupffer cells, ultimately suppressing the NF-κB pathway, reducing Kupffer cell activity, and decreasing the secretion of inflammatory factors. We hypothesized that SCFAs, as metabolites of gut microbes, could be involved in the regulation of liver immune activity in the AH-like environment through the LPS-TLR4-NF-κB pathway, without further experimental proof, of course [100].

In vitro experiments investigating non-alcoholic liver injury have shed light on the beneficial effects of certain secretions from intestinal bacteria in alleviating hepatic inflammation and oxidative stress. Tayyeb et al. conducted a study in which HepG2 cells were treated with four SCFAs; namely, propionate, butyrate, and valerate. They observed a negative correlation between the expression of NF-κB and PPAR-α. It was postulated that SCFAs might exert their inhibitory effects on NF-κB expression by activating PPAR-α, thereby mitigating the inflammatory response [111].

Studies have demonstrated that the Nrf2 pathway holds promise for the treatment of ALD and fibrosis, which is also a serious pathological injury of AH, by addressing oxidative stress. For instance, Ishida et al. utilized sulforaphane to treat HepaRG cells and observed an upregulation of HMOX1, NQO1, and GSTM3 gene expression, indicating an antioxidant response mediated by Nrf2 upregulation [138]. Moreover, emerging evidence suggests the involvement of gut microbes in regulating this pathway. Lactobacilli, a type of Firmicute, includes the human commensal Lactobacillus rhamnosus GG. Bejan et al. demonstrated that this particular strain of Lactobacillus stimulates the Nrf2 pathway in the liver, leading to antioxidative responses. Unlike the more common Firmicutes or some of the intestinal microbiotas in the Bacteroidetes that are involved in the regulation of liver physiology by secreting SCFAs, Lactobacillus rhamnosus GG produces a small molecule called the 5-methoxyindoleacetic acid (5-MIAA) molecule. When 5-MIAA is transferred from the intestine to the liver, it activates Nrf2 signaling in hepatocytes to enhance cellular antioxidant responses [112,139].

The inhibition of inflammation and reduction of oxidative stress damage in the liver is a more direct and rapid way to ameliorate liver damage, with great target potential. If the appropriate flora and pathways can be specifically targeted, it may be possible to inhibit the acute development and chronic inflammatory state of alcoholic hepatitis more effectively.

#### 4.2.4. Liver Regeneration

In AH, the gut microbiota can also impact the life cycle of hepatocytes, including liver regeneration. The process of liver regeneration is intricate and influenced by various factors, including metabolism and immunity mediated by the intestinal microbiota.

The regulation of liver regeneration by the gut microbiota often involves cytokine-dependent mechanisms. For instance, interleukin 6 (IL-6) binds to its receptors on target cells, forming a complex that further binds to glycoprotein 130 (gp130 receptor). This binding activates the JAK/STAT pathway and downstream genes. The activated STAT3 enters the nucleus and activates genes associated with mitosis in hepatocytes, leading to the transition of quiescent cells into the mitotic process [140,141]. Additionally, IL-6 can upregulate the expression of the anti-apoptotic protein Mcl-1L through the activation of the JAK/PI3K/Akt/CREB pathway, thereby controlling hepatocyte apoptosis [142]. These processes are beneficial for liver regeneration and recovery.

Certain intestinal microbiota found in the colon and ileum, such as Clostridium and Eubacterium, play a role in BA synthesis and contribute to controlling the interaction of BAs with hepatocytes [143,144]. The effects of BAs on the liver are dose-dependent, with excessive levels of BAs being toxic, while low levels of BAs stimulate short-term liver regeneration. Therefore, the synthesis rate of BAs needs to be regulated. Following liver damage, the BA levels may rise, leading to the activation of the FXR, which is highly expressed in enterocytes and hepatocytes. This activation results in an upregulation of fibroblast growth factor 15 (FGF-15) production and its entry into the liver through the portal system. FGF-15 then inhibits the expression of CYP7A1 via the FGFR receptor on hepatocytes, thereby limiting the rate of BA synthesis [145,146,147].

Clostridium butyricum can influence the apoptosis of liver cells to maintain normal liver physiology and a healthy state by suppressing the gene expression of BAX, which is followed by the secretion of SCFAs [148]. The stability of Firmicutes in the small intestine is also essential for the regenerative function of the liver. A decreased abundance of Firmicutes can lead to defects in liver regeneration [149].

It should also be noted that intestinal microbiotas can regulate the process of hepatocyte regeneration by affecting the synthesis of cellular structural materials. Yin, Y. et al. treated 70% of hepatectomized mice with antibiotic and bacterial colonization of SCFAs-producing microbiotas and found that the abundance of Proteobacteria in the intestinal lumen of the mice whose intestinal microbiota composition was impaired by antibiotic treatment was significantly increased, the synthesis of SCFAs was significantly reduced, and the expression of lipogenic enzyme SCD1 was delayed. Additionally, the rate of liver regeneration and survival rate were both significantly decreased. In contrast, mice in the SCFAs-producing colony-colonized group showed a greater increase in hepatocyte membrane phospholipid synthesis, cell regeneration, and survival rate. Thus, intestinal microbiotas are necessary for liver regeneration. SCFAs induce upregulation of hepatic lipogenic enzyme SCD1 expression and promote hepatic membrane phospholipid synthesis, which accelerates hepatocyte regeneration and proliferation [19].

In addition to the antioxidative stress function mentioned earlier, activation of the Nrf2 pathway by intestinal flora, such as Lactobacilli, presents a promising therapeutic approach to promote liver regeneration. In a pharmacological stimulation assay targeting Nrf2, wild-type mice that underwent partial hepatectomy and were treated with bardoxolone methyl exhibited a higher rate of liver volume recovery compared to Nrf2 knockout mice undergoing the same operation. This effect may be closely associated with the oxidative stress and inflammation-suppressing effects in the immune system attributed to Nrf2 activation. Further exploration of this relationship is warranted [112,150].

#### 4.2.5. Intestinal Microbiota as Target

The gut microbiota and its products have predominantly been discussed in terms of therapeutic or intervention applications. However, a deeper understanding of their function as targets is needed. Current clinical studies have primarily focused on broad approaches such as FMT and probiotic prebiotics, but they have not been able to precisely target the microbiome. Utilizing high-throughput gene sequencing, genomics, and metabolomics may shed light on a series of alterations in the gut microbiota with increasing clarity.

Xiang et al. conducted a study in which alcoholic hepatitis mice were treated with Schisandra chinensis extract (SCE), and the changes in the gut microbiota were analyzed using high-throughput sequencing based on 16S rRNA. Following SCE treatment, the proliferative activity of SCFAs-producing bacteria, namely Lactobacillus and Bifidobacterium, was more pronounced compared to the control group. In contrast, the proliferation of pathogenic Escherichia-Shigella bacteria was inhibited. Additionally, SCE significantly reduced liver lipid accumulation and effectively suppressed the elevation of serum ALT and AST levels. Furthermore, the study measured the decrease in superoxide dismutase (SOD) activity and the increase in inducible nitric oxide synthase (iNOS) activity in mice, which led to the suppression of hepatic inflammatory factors and Cyp2e1 expression, consequently reducing ROS levels and blocking their production. Oxidative stress, nitrosative stress, hepatic inflammation, and intestinal barrier function were improved in alcoholic hepatitis mice [118]. Another study by Xu et al. demonstrated that oenthein B (OEB) supplementation in alcoholic hepatitis-related diseased mice exhibited antioxidative stress and anti-inflammatory effects by modulating the Keap1/Nrf2 and TLR4/NF-κB signaling pathways. Interestingly, OEB was also able to effectively regulate the composition of the intestinal microbiota, such as increasing the abundance of SCFAs-producing bacteria like Muribaculaceae and Erysipelotrichaceae while inhibiting the proliferation of Gram-negative bacteria like Akkermansia, thus improving the intestinal ecology. Increased synthesis of SCFAs may activate the antioxidant Nrf2 signaling pathway in the liver, while the reduction in lipopolysaccharide (LPS) levels due to the decrease in Gram-negative bacteria prevented the development of inflammation [119]. Inulin, a natural prebiotic, also showed potential in ameliorating alcoholic hepatitis-related diseases. Mice treated with inulin exhibited reduced levels of hepatic inflammatory cytokines such as TNF-a, IL-6, and IL-17A. Moreover, the membrane potential of TLR4 receptors was significantly lower compared to the control group, indicating the inhibition of TLR4 receptors. Among the four dominant bacterial groups in the alcohol/inulin group, Proteobacteria and Actinobacteria displayed more noticeable changes, while Firmicutes and Bacteroidetes seemed to be less affected. In other words, inulin has the potential to improve alcoholic liver disease (ALD) in mice by inhibiting the LPS-TLR pathway and modulating the intestinal microbiota [120]. Precision-targeted therapy and colony editing of the gut microbiota also hold promise and research value. Duan et al. found that cytolysin, a toxin secreted by *Enterococcus faecalis*, plays a crucial role in alcohol-induced liver injury. Through the use of bacteriophages specifically targeting humanized mice colonized with intestinal microbiota from alcoholic hepatitis patients, a more significant reduction in cytolysin levels in the livers of the mice was observed, resulting in pronounced improvement in liver injuries [44].

In the exploration of the therapeutic value of the gut microbiota, this not only serves as a research subject but also as a tool to facilitate research development. For instance, genetically engineered intestinal bacteria can be employed in studying the fundamental mechanisms of alcoholic hepatitis [56]. Although targeted therapies for the gut microbiota still require extensive clinical trials to validate their relevance in humans and their efficacy in patients with alcoholic hepatitis, they hold great promise in the future of research demand.

## 5. Conclusions

As a progressive and severe stage of ALD, AH often shows a poor prognosis and a high mortality rate in the short term. Evidence indicates that alcohol consumption, aging, and genetic factors can all promote the occurrence of AH. Additionally, more and more research has determined that the intestinal microbiota plays a crucial role in the etiology of AH. Subsequently, the broken intestinal barrier caused by alcohol can promote the translocation of gut microbiota to the liver. Moreover, the immune response triggered by the gut microbiota in the liver can cause inflammation which further increases the damage to the liver. In addition, the metabolism of hepatocytes and gut microbiota can be affected in the progression of AH. Further, some abnormalities in the products can be detected for diagnosis. LPS which is produced by the gut flora can also contribute to the inflammation in the liver by binding to the TLR4. Cytokines such as IL-20 and TNF-α are all confirmed to participate in the development of AH through different pathways, whereas different types of cytokines may play different roles in the procession of AH. In the current treatment, prednisolone is the most useful treatment for severe AH. Liver transplantation has also been chosen to treat patients who are not sensitive to prednisolone. However, the prognosis for these treatment modalities is generally unsatisfactory, and more treatment options need to be explored. Specific gut microbiota like cytolytic *Enterococcus faecalis* have shown potential as therapeutic targets for the clinical treatment of AH [44]. Supplementation with therapeutic substances such as vitamins in combination with other medications has been shown to be more effective. As gut microbes play an important role in the progression of AH, understanding the specific role of the intestinal microbiota in AH is essential for both understanding the disease’s pathogenesis and identifying potential treatment strategies. Targeting gut microbes has positively contributed to the treatment of AH in many animal and human cell experiments, yet microbial therapies are still lacking in clinical application. This paper provides a review of the involvement of the intestinal microbiota in the development of AH and highlights potential targets that have demonstrated utility in AH treatment.

## Figures and Tables

**Table 1:** Clinical studies of intestinal microbiotas in AH and related diseases.

Intervention	Design	Result
*Lacticaseibacillus rhamnosus* R0011 and *Lactobacillus helveticus* R0052 [93]	Total: 100 AH patients without severe liver damage. Group A: 7 days of cultured *L. rhamnosus* R0011 and *L. helveticus* R0052 treatment (*n* = 44). Group B: 7 days of placebo treatment (*n* = 45).	LPS, ALT, and γ-GTP levels were significantly reduced in the probiotic treated group; The percentage of Bacteroidetes increased and the percentage of Proteobacteria and Fusobacteria decreased.
Bifidobacterium bifidum, *Lactobacillus plantarum* 8PA3 [94]	Total: 66 patients with alcoholic psychosis (26 mild AH). Group A: 5 days of *Bifidobacterium bifidum* and *Lactobacillus plantarum* 8PA3 treatment (*n* = 32). Group B: 5 days of Standard therapy (abstinence and vitamins, *n* = 34). Group C: healthy control group without alcohol consumption (*n* = 24).	Levels of ALT, AST, GGT, LDH, and TBIL decreased significantly after treatment in patients with mild AH; Activities of AST and ALT decreased and gut microbiota’s composition changed compared to standard treatment; Abundance of probiotics in the probiotic treatment group increased.
*Lactobacillus casei* Shirota [95]	Total: 33 subjects included. Group A: 28 days of *Lactobacillus casei* Shirota treatment (*n* = 12). Group B: Patients not receiving probiotics (*n* = 8). Group C: Healthy control group (*n* = 13).	TNFR1, sTNFR2, and IL10 levels were increased, the expression of TLR4 was restored, and the function of neutrophils was restored
Fecal microbiota transplantation [96]	Total: 26 severe AH patients. Group A: 7 days of FMT treatment through the nasoduodenal tube (*n* = 12). Group B: Standard treatment (*n* = 18).	Improvement in serious indicators of liver disease and improvement in serious complications; Changes in the composition of the flora, with a decrease in Proteobacteria and an increase in Actinobacteria; Significantly improved survival in the FMT group (87.5% vs. 33.3%).
Fecal microbiota transplantation [97]	Total: 120 steroid-eligible severe AH patients Group A: 7 days of FMT treatment through the nasoduodenal tube (*n* = 60). Group B: 7 days of prednisolone treatment for 40 mg/d, If Lille score < 0.45, continued with the same dose to 28 days, otherwise continued with nutritional management, antibiotics, and supportive care (*n* = 60).	The 28- and 90-day survival rates were significantly higher in the FMT group than in the prednisolone treatment (88.33% vs. 78.33% and 75% vs. 56.6%); The FMT group exhibited a lower infection rate; Decrease in pathogenic taxa and increase in Alphaproteobacteria and Thaumarcheota in FMT flora composition.

**Table 2:** Therapeutic potential of intestinal microbiotas and products in AH-related diseases.

Potential	Intervention	Mechanism	Effect
Intestinal barrier protection [99,100,101,102]	Butyrate	Activates AMPK pathway to promote ZO-1 and occludin migration and assembly.	Increases tight junction assembly.
	Propionate	Upregulates the expression of epithelial claudin-1, occludin, ZO-1, E-cadherin, MUC2, Reg 3β, and Reg 3γ.	Promotes tight junction synthesis, restores gut mucosa, and suppresses gut inflammation.
	*Lactobacillus plantarum* *Lactobacillus acidophilus*	Upregulates tight junction proteins expression, reduces serum LPS, and promotes SCFAs secretion.	Restores intestinal epithelial permeability.
	Bacteroides thetaiotaomicron	Suppresses MUC1 transcription and upregulates MUC2, GLP-1, and FGF-15 expression.	Restoring intestinal mucosal function and eubiosis.
Metabolic regulation [103,104,105,106,107,108,109]	Butyrate	Suppresses HDAC3l to promote FGF-21 expression.	Promotes lipid oxidation and ketogenesis.
		Activates PPAR-α ligand to induce FGF-21 expression.	Promotes fatty acid oxidation and reduces fatty acid synthesis.
		Suppresses PPAR-γ expression to activate UCP2-AMPK-ACC pathway.	Promotes hepatic lipid β-oxidation, improves insulin resistance.
	Propionate	Activates IGN and stimulates PYY and GLP-1 synthesis.	Activates intestinal gluconeogenesis and improves insulin sensitivity.
	Acetate	Activates the GPR43-AKT-GSK3 signaling pathway.	Promotes glycogen metabolism in hepatocytes.
	Lactobacillus plantarum ZY08	Reverses gene expression of Cd36, Dgat1, Dgat, and Fasn. Increases the expression of RRAR-α.	Reduces the level of hepatic steatosis and fat accumulation.
Inflammation and oxidative stress protection [110,111,112,113,114,115]	Butyrate	Inhibits TNF-α activation to suppress NF-κB pathway.	Inhibits inflammation.
	Propionate	Inhibits neutrophils synthesizing TNF-α,CINC-2αβ, NO, and ROS.	Reduces hepatic inflammation and oxidative stress injury.
	Lactobacillus rhamnosus GG	Produces 5-MIAA to activate Nrf2 signaling.	Promotes hepatocytic antioxidant response.
	Bifidobacterium longum R0175	Produces sedanolide to activate Nrf2 signaling.	Reduces hepatic inflammation and oxidative stress injury.
Liver regeneration [19,116,117]	Lactiplantibacillus plantarum AR113	Upregulates TNF-α, HGF, TGF-β expression to activate NF-κB signaling.	Initiates the liver regeneration process.
	Butyrate Propionate Acetate	Induces upregulation of hepatic lipogenic enzyme SCD1 expression.	Promotes hepatic membrane phospholipid synthesis to accelerate hepatocyte regeneration and proliferation.
	Listeria monocytogenes	Produces InlB321/15 to activate the HGFR-dependent MAPK signaling pathway.	Stimulated liver regeneration
Targeted modulation [44,118,119,120]	Schisandra chinensis extract	Increases iNOS activity and inhibits the activity of SOD and CYP2E1, increases Lactobacillus and Bifidobacterium activity	Reduces lipid accumulation, improves oxidative stress, nitrifying stress, and liver inflammation, and restores intestinal barrier function.
	Oenthein B	Increases Muribaculaceae and Erysipelotrichaceae abundance, decreases Akkermansia abundance, activates the Keap1/Nrf2 pathway, and inhibits the TLR4/NF-κB pathway.	Inhibits oxidative stress and inflammation and improves intestinal ecology.
	Inulin	Inhibits the LPS-TLR4-Mψ axis and regulates intestinal microbiotas.	Reduces liver inflammation and restores intestinal dysbiosis.
	Bacteriophages	Specifically targets cytolytic *Enterococcus faecalis* and re-edits the gut microbiota to reduce cytolysin.	Alleviates AH-related liver damage.

## Data Availability

Not applicable.
